# Evasion of the Host Immune Response by Betaherpesviruses

**DOI:** 10.3390/ijms22147503

**Published:** 2021-07-13

**Authors:** Daniel G. Sausen, Kirstin M. Reed, Maimoona S. Bhutta, Elisa S. Gallo, Ronen Borenstein

**Affiliations:** 1 Department of Microbiology and Molecular Cell Biology, Eastern Virginia Medical School, Norfolk, VA 23507, USA; SausenDG@EVMS.EDU (D.G.S.); ReedKM@EVMS.EDU (K.M.R.); BhuttaM@EVMS.EDU (M.S.B.); 2 Board-Certified Dermatologist and Independent Researcher, Norfolk, VA 23507, USA; esgallomd@hotmail.com

**Keywords:** betaherpesvirus, immune evasion, viral evasion, immune response, HCMV, HHV-6A, HHV-6B, HHV-7

## Abstract

The human immune system boasts a diverse array of strategies for recognizing and eradicating invading pathogens. Human betaherpesviruses, a highly prevalent subfamily of viruses, include human cytomegalovirus (HCMV), human herpesvirus (HHV) 6A, HHV-6B, and HHV-7. These viruses have evolved numerous mechanisms for evading the host response. In this review, we will highlight the complex interplay between betaherpesviruses and the human immune response, focusing on protein function. We will explore methods by which the immune system first responds to betaherpesvirus infection as well as mechanisms by which viruses subvert normal cellular functions to evade the immune system and facilitate viral latency, persistence, and reactivation. Lastly, we will briefly discuss recent advances in vaccine technology targeting betaherpesviruses. This review aims to further elucidate the dynamic interactions between betaherpesviruses and the human immune system.

## 1. Introduction

Betaherpesviruses, a widespread subfamily of viruses within the herpesviridae family, are nearly ubiquitous in the global population [1,2,3] and include human cytomegalovirus (HCMV), human herpesvirus (HHV) 6A, HHV-6B, and HHV-7 [4].

Like other herpesviridae, betaherpesviruses are comprised of four primary sections: the double-stranded viral DNA, the capsid, the tegument, and the envelope. The capsid surrounds the DNA core, while the tegument is a protein-filled area surrounding the capsid. The envelope, the most exterior structural component of herpesviridae, is studded with glycoprotein spikes [4,5]. Tegument proteins are involved in a wide variety of viral activities, including virion assembly, gene expression, egress, and immune evasion [6,7,8]. Envelope glycoproteins play a particularly prominent role in protection and in mediating viral entry [5,9,10].

The tropism of HCMV [11,12,13], HHV-6 [14,15,16,17], and HHV-7 [17] has been reviewed in depth elsewhere. Specific cellular receptors and viral proteins play an important role in the determination of viral tropism and are also detailed in the aforementioned reviews. HCMV can infect a variety of different cell types in vivo and in vitro, including epithelial cells, endothelial cells, fibroblasts, and smooth muscle cells [18]. While many receptors contribute to viral entry in HCMV, there are two models of viral entry, one for fibroblasts and one for epithelial and endothelial cells [13]. In fibroblasts, the cellular platelet-derived growth factor-α receptor (PDGFRα) interacts with the HCMV gH/gL/gO trimer complex (TC), which activates glycoprotein B (gB) to fuse the viral envelope with the cell membrane [13]. In epithelial and endothelial cells, the cellular receptor neuropilin2 (Nrp2) interacts with the HCMV gH/gL/pUL128L pentamer complex (PC) to stimulate viral endocytosis followed by TC activation of gB, which facilitates viral entry [13]. Once viral entry is accomplished, local spread is mediated by direct cell-to-cell transmission [13]. However, HCMV primarily establishes latency in cells arising from myeloid progenitors. HCMV has been found to infect dendritic cells latently as well as peripheral mononuclear blood cells, including monocytes and CD34+ progenitor cells [11]. Infection begins following exposure to contaminated saliva, mucosa, skin lesions, or genital secretions [1]. HCMV disease associations include transplant rejection, retinitis, and serious birth defects [19,20,21]. HCMV can also present as a mono-like illness [22].

HHV-6 also infects a broad range of cells in vivo and in vitro, although it preferentially infects activated CD4+ T lymphocytes. In vivo, HHV-6 can infect tissues ranging from the brain to the liver [14] as well as a variety of additional cell types including endothelial cells, NK cells, and myeloid cells [23,24]. HHV 6 is composed of two related but distinct viruses, HHV-6A and HHV-6B [25]. HHV-6A but not HHV-6B has been shown to infect endometrial cells [26]. However, HHV-6A and HHV-6B differ in their replicative ability within specific transformed T-lymphocyte cell lines [15,16]. For example, HHV-6A is most commonly maintained in HSB-2 or JJhan T cell lines while HHV-6B is preferentially maintained in Molt3 or MT4 [15,23]. In addition, a recent study showed that HHV-6A_GS_ was capable of transcribing the viral genes U7 and U23 in the T cell lines Peer and Jurkat while HHV-6B_PL1_ could not. Both HHV-6A and HHV-6B infect Molt3 and SupT1 T cells [27]. HHV-6A uses the cell surface receptor CD46 to mediate viral entry, while HHV-6B uses CD134 [14,17]. HHV-7 has similar tropism to HHV-6, selectively infecting CD4+ T cells. However, HHV-7 can be observed in various tissues in vivo, including the skin, salivary glands, and other organs [17]. Like HCMV, contaminated saliva, mucosa, skin lesions, or genital secretions leads to HHV-6 and HHV-7 infection [1]. The primary disease associated with HHV-6 is exanthem subitum, commonly known as roseola or sixth disease, a self-limited disease seen in infants aged 6 months to 2 years of age [14]. More serious disease associations include seizures, encephalitis/encephalopathy, retinitis, and hepatitis [14,28]. Complications including graft versus host disease and lower respiratory tract infections have been reported in the setting of organ transplantation [29]. HHV-6 has also been associated with neurodegenerative disorders such as multiple sclerosis [30,31] and Alzheimer’s disease [32], although a definitive link has yet to be established. HHV-7 is a sparsely researched virus with limited disease associations at this time, although it is also thought to cause exanthem subitum [17]. It, too, has been associated with encephalitis/encephalopathy [28] and more tenuously with Alzheimer’s disease [32]. Betaherpesviruses have also been associated with multiple sclerosis [33,34,35,36] and chronic fatigue syndrome [37,38], although these associations have been questioned in the past [39,40,41].

To successfully establish an infection, betaherpesviruses must first avoid the sophisticated human immune system. In fact, betaherpesviruses have evolved an array of methods for just this purpose. Their high seroprevalence and ability to establish lifelong latent infections [4] are testaments to the success of their immunoevasive techniques. This review aims to illustrate the complex interplay between betaherpesviruses and the immune system, with a greater emphasis placed on HCMV due to the relative abundance of new research focusing on this virus. It will explore DNA sensors responsible for detecting the initial infection within a cell as well as interactions between betaherpesviruses and immune effector cells, specifically natural killer cells and T cells. It will also examine the control of latency and reactivation and will conclude with a brief discussion on recent vaccination efforts targeting betaherpesviruses. Betaherpesvirus immunoevasive strategies will be discussed throughout to better highlight the interactions between the human immune system and betaherpesviruses.

## 2. Innate Immune Response Pathways

**Figure 1:**
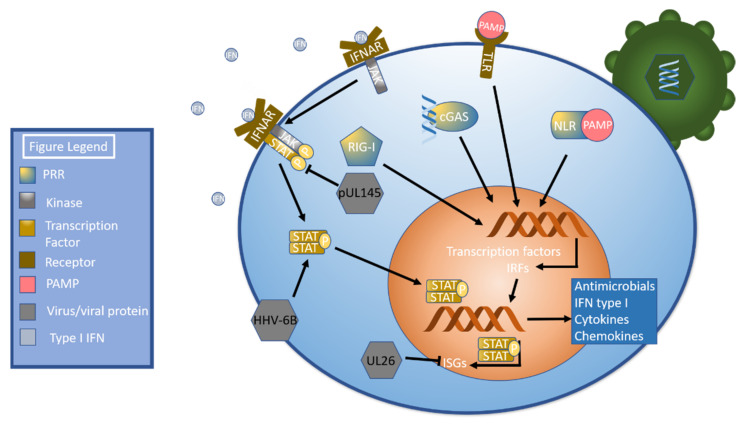
Pathogen Sensing, Interferon (IFN), and the JAK-STAT Pathway. Pathogen invasion triggers several pattern recognition receptors (PRRs), including NOD-like receptors (NLRs), RIG-I -like receptors (RIG-I), cGAS, and TLRs (toll-like receptors). These PRRs activate signal transduction pathways that culminate in the production of transcription factors and interferon regulatory factors, which in turn leads to the production of antimicrobial peptides, proinflammatory cytokines, chemokines, and type I IFN. Type I IFN then binds to the interferon α receptor (IFNAR), which phosphorylates JAK. Phosphorylated JAK recruits STAT, which is then phosphorylated, dimerizes, and translocates to the nucleus. Once in the nucleus, STAT upregulates the transcription of ISGs. The HCMV protein pUL145 inhibits STAT, while HHV-6B has been shown to upregulate STAT. The HCMV protein UL26 inhibits ISGs and ISGylation.

A primary mechanism to induce the innate immune response begins when pathogen associated molecular patterns (PAMPs) are detected by pattern recognition receptors (PRRs), including toll-like receptors (TLRs), NOD-like receptors (NLRs), RIG-I-like receptors, and cGAS-STING [42,43]. Pattern recognition receptor activation stimulates signal transduction pathways that culminate in the activation of transcription factors including NF-κB and interferon regulatory factors. These transcription factors drive the production of antimicrobial peptides, proinflammatory cytokines, chemokines, and type I interferons [42]. Type I Interferon (IFN) binds the interferon α receptor (IFNAR), which initiates the janus kinase-signal transducer and activator of transcription (JAK-STAT) pathway [44] by phosphorylating JAK [45]. Phosphorylated JAK recruits STAT, a transcription factor [44], which is then phosphorylated and dimerizes. Dimerized STAT translocates to the nucleus [45], which leads to the production of interferon stimulated genes, or ISGs [44] (Figure 1). Betaherpesviruses have developed numerous methods of evading this pathway as will be discussed shortly.

### 2.1. The cGAS-cGAMP-STING Signaling Pathway

**Figure 2:**
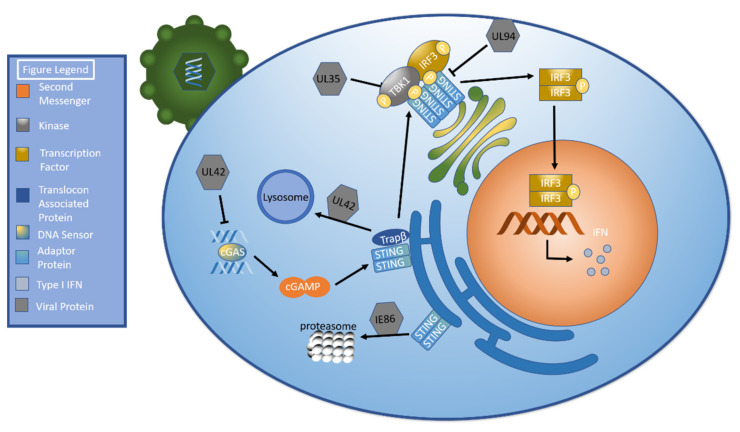
The cGAS-cGAMP-STING Pathway and Human Cytomegalovirus Immunoevasive Methods. The cGAS-cGAMP-STING pathway is activated after cGAS detects abnormal double-stranded DNA in the cytosol. cGAS produces cGAMP, which stimulates the translocation of STING to the golgi, where it oligomerizes. STING recruits TBK1, which then autophosphorylates and phosphorylates STING. STING recruits IRF3 to create a STING/TBK1/IRF3 complex. TBK1 then phosphorylates IRF3, which dissociates from the complex, dimerizes, and translocates to the nucleus. Once there, it stimulates interferon transcription. UL42 inhibits cGAS and promotes TRAPβ degradation via lysosomes. UL94 inhibits STING dimerization and prevents STING from recruiting TBK1. UL35 inhibits TBK1. IE86 facilitates the proteasomal degradation of STING.

Cyclic GMP-AMP synthase (cGAS) is a PRR that senses cytosolic double stranded DNA. cGAS stimulation leads to the creation of a cyclic GMP-AMP (cGAMP) dinucleotide that activates stimulator of interferon genes (STING) [46], a dimeric transmembrane protein [47] primarily expressed in the endoplasmic reticulum [48]. cGAS binding stimulates the translocation of STING to post-golgi compartments [47,49], where it oligomerizes [47]. Tank binding kinase 1 (TBK1) is recruited to STING [50] and activated by autophosphorylation [51]. TBK1 subsequently phosphorylates STING [52], which recruits the transcription factor interferon regulatory factor 3 (IRF3) to create a STING/TBK1/IRF3 complex. This allows TBK1 to phosphorylate IRF3, which then dissociates from the complex and dimerizes [50]. The dimer translocates to the nucleus, where it forms a complex that induces IFN transcription [53] (Figure 2). An excellent review by Cai et al., describes the cGAS-cGAMP-STING pathway [54].

Perhaps unsurprisingly, HCMV has developed several methods to evade this pathway. A recent study found that human foreskin fibroblast (HFF) cells infected with a HCMV UL42-defective virus mounted a stronger immune response to HCMV than wild type virus. Infection with a UL42-defective virus resulted in higher levels of mRNA transcribed from the IFNB1, ISG56, CXCL10, and IL6 genes. There was also increased phosphorylation of STING, TBK1, and IRF3. Subsequent studies showed that UL42 interferes with the normal function of both cGAS and STING. UL42 inhibits the ability of cGAS to bind HCMV DNA as well as its ability to oligomerize. It impedes STING trafficking by promoting the degradation of TRAPβ, which had previously been shown to be essential in STING trafficking [55], via an autophagic lysosomal pathway. Lastly, UL42 synergistically inhibits the cGAS-cGAMP-STING pathway with UL31 and UL82 [56].

HCMV UL94 is another tegument protein that targets STING to evade the innate immune response. Indeed, UL94 overexpression in HEK293T cells resulted in decreased IFNB1, CXCL10, and RANTES transcription but failed to significantly inhibit protein products from other immune pathways, including IFN-γ and tumor necrosis factor alpha (TNF-α). UL94 downregulated antiviral genes in the presence of UV-treated HCMV, which can infect cells but is unable to transcribe or translate genetic material. While UL94 increased HCMV replication in control cells, it did not augment replication in STING-deficient cells. It was shown to exert this inhibitory effect by inhibiting STING dimerization, which in turn prevents appropriate STING trafficking to perinuclear microsomes. It also prevents TBK1 from complexing with STING [57].

Immediate early genes represent another modality employed by HCMV to disrupt the cGAS-cGAMP-STING pathway. IE86 has been shown to stimulate STING degradation via proteasomes [58]. Recent experiments by Lee et al., used a luciferase reporter assay to determine STING-firefly fusion protein levels in the presence of wild type and mutated IE86. Mutants that were still capable of downregulating STING contained amino acids 136–289 [59].

The phosphoprotein UL35 represents another mechanism utilized by HCMV to downregulate IFNβ transcription, in this case by interfering with TBK1. Cotransfection of TBK1 and UL35 in HEK293T cells resulted in decreased phosphorylation of both TBK1 and IRF3. IRF3-5D, a constitutively active form of IRF, did not exhibit decreased activity in the presence of UL35. Notably, signaling through both cGAS-cGAMP-STING and retinoic acid inducible gene I (RIG-I), an RNA sensing PRR [60], was inhibited [61].

The interactions between PRRs and HHV-6A, HHV-6B, and HHV-7 have not received the same amount of attention as that between PRRs and HCMV. However, Bortolotti et al., did run a series of experiments examining the effects of these three viruses on DNA sensor signaling and their downstream molecules in natural killer (NK) cells. Interestingly, while HHV-6A increases expression of STING mRNA during infection of NK92 cells, there was no corresponding increase in STING protein levels. STAT6 phosphorylation was also increased. HHV-6B infection did not alter STING mRNA expression or STAT6 phosphorylation. While cGAS expression was not affected during HHV-6A or -6B infection, decreased interferon gamma inducible protein 16 (IFI16) levels were noted in both HHV-6A and HHV-6B-infected cells. TBK1 levels appeared to be increased in all three viruses examined, although the authors were concerned about potential cross-reactivity between the TBK1 autoantibody used in the Western blot and other HHV proteins. Aberrant STING/STAT activation was further demonstrated in HHV-6A infection by their atypical colocalization to the peri-nuclear/cytoplasmic region. In contrast, these molecules primarily remained in the cytosol in control NK cells [62].

HHV-7 infection apparently has a relatively minor influence on the cGAS-cGAMP-STING pathway. There was a small decrease in cGAS expression and the previously mentioned increase in TBK1 protein expression. No other examined expression levels were affected by HHV-7 [62].

### 2.2. Toll-Like Receptors

**Figure 3:**
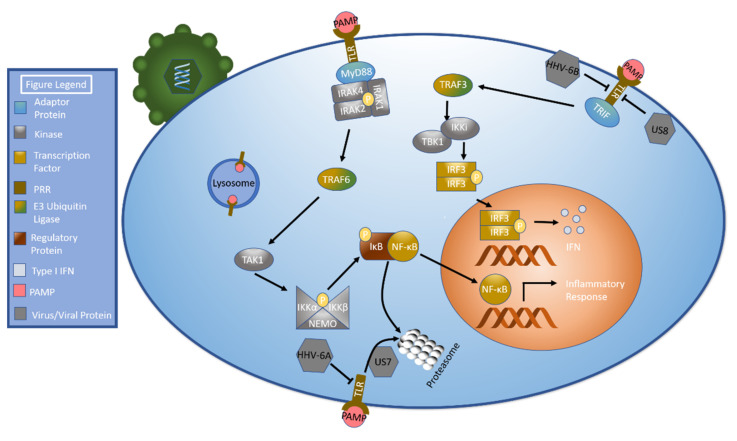
Toll-Like Receptor (TLR) Signaling and Inhibition. The TLR signaling pathway begins after a pathogen-associated molecular pattern (PAMP) binds to the TLR. There are two main signaling pathways activated by TLRs. TLR activation through MyD88 results in the formation of a myddosome, which includes MyD88, IRAK1, IRAK2, and IRAK4. The myddosome then activates TRAF6, which activates TAK1. TAK1 phosphorylates the IKK complex, which in turn phosphorylates IκB. IκB phosphorylation triggers its proteasomal degradation and the subsequent release of the transcription factor NF-κB. The second pathway involves TRIF associating with the TLR. TRIF activates TRAF3, which activates TBK1 and IKKi. These phosphorylate IRF3. As in the STING pathway, IRF3 dimerizes and translocates to the nucleus to induce an interferon response. The HCMV protein US7 inhibits signaling by targeting TLR3 and TLR4 for proteasomal degradation, while the HCMV protein US8 inhibits signaling by destabilizing TLR3 and TLR4. Of note, HHV-6A and HHV-6B have been shown to downregulate TLR9 protein levels.

Toll-like receptors are a membrane-bound class of PRRs capable of sensing numerous PAMPs including lipids, lipoproteins, proteins, and nucleic acids. TLRs can be found in the plasma membrane as well as in the membranes of organelles such as endosomes, lysosomes, and endolysosomes [63]. Upon activation, TLR complexes with multiple IL-1R associated kinases (IRAK) and the inflammatory signaling adaptor protein MyD88 in a structure called the myddosome [64,65]. IRAK4 is activated by autophosphorylation, which leads to the activation of IRAK1 and IRAK2. This leads to the activation of the E3 ubiquitin ligase TNF receptor-associated factor 6 (TRAF6), a key intermediate in TLR signaling [65]. TRAF6 activates the serine/threonine kinase transforming growth factor-β activated kinase-1 (TAK1). This, in turn, activates several downstream molecules, including activating the I-kappa kinase (IKK) complex comprised of IKKα, IKKβ, and NF-κB essential modulator (NEMO, also called IKK-γ) by phosphorylation [65,66]. The IKK complex phosphorylates IκB proteins. This triggers the proteasomal degradation of IκB, which releases NF-κB [67]. NF-κB translocates to the nucleus, where it helps orchestrate the inflammatory response [68]. Notably, TLRs can signal through an alternate pathway in which TIR-domain-containing adapter-inducing interferon-β (TRIF) complexes with TRAF3. TRAF3 can subsequently activate TBK1 and IKKi, which in turn phosphorylate IRF3 [67]. This pathway is demonstrated in Figure 3.

Two HCMV-expressed proteins, US7 and US8, have been implicated in the inhibition of the toll-like receptors TLR3 and TLR4. HFF cells were made to express US7 or US8 and then challenged with double-stranded DNA. These cells demonstrated significantly decreased expression levels of numerous immunomodulatory genes relative to control cells with the largest reductions noted in IFNβ, TNFSF10, CCL8, CXCL10, CXCL11, IFIT3, and ISG. HFF cells expressing US7 or US8 were subsequently exposed to synthetic dsRNA (poly(I:C)) and LPS, which activate TLR3 and TLR4, respectively. The cells’ ability to activate the TLR3 or TLR4 signaling pathways was disrupted as evidenced by decreased IFNβ, CXCL10, and CXCL11 transcription. Suppression was noted in a variety of cell lines, including HEK293T cells and monocyte/macrophage lineage cells. US7 and US8 were both shown to decrease TLR3 and TLR4 production but not mRNA expression. Subsequent studies showed that US7 degrades TLR3 and TLR4 via a ubiquitin/proteasome system while US8 destabilizes TLR3 and TLR4. Consistent with these results, TLR3 and TLR4 protein expression and subsequent IFNβ levels were knocked down in HFF cells infected with wild type virus compared to those infected by HCMV with a deletion of the US7-16 region [69].

HHV-6 has previously been shown to diminish TLR signaling [70]. A more recent study demonstrated that mRNA and protein levels of TLR9 were decreased in HHV-6A infection. HHV-6B did not affect mRNA levels of TLR9 but did decrease protein expression. MyD88 expression, which is important in TLR9 signal transduction [63], was unchanged in both HHV-6A and HHV-6B infection. HHV-7 did not affect mRNA or protein levels of TLR9. MyD88 expression was also unaffected. Consistent with these results, infection with HHV-6B and HHV-7 increased TNFα, IFNα, and IL8 expression while HHV-6A infection only caused a slight increase in TNFα and IL8 expression [62].

### 2.3. Interferon Response and Inhibition

microRNAs (miRNAs) are increasingly recognized as post-transcriptional modifiers with roles in a variety of physiological and disease processes [71]. Immune regulation is one such function, and recent research has begun to uncover the role of miRNAs in establishing an IFN response against HCMV. miR-182 is one such miRNA recently proven to counteract HCMV infection by modulating the IFN response. In fact, inhibiting miR-182 reduced the IFN response to HCMV. miR-182 overexpression reduced viral copies, an effect that was attenuated in IFNAR knockdown cells. This is accomplished by targeting forkhead box O 3 (FOXO3), thereby decreasing its quantity. The authors also noted that transfection with a miR-182 mimic resulted in elevated levels of IRF7 in HCMV-infected cells. Of note, FOXO3 was previously shown to block IRF7 expression [72]. In essence, miR-182 targets FOXO3 to prevent FOXO3 mediated IFN7 downregulation [73].

miR-221 is upregulated in HCMV infections and, like miR-182, works by augmenting the IFN response. However, unlike miR-182, miR-221 targets suppressor of cytokine signaling 1 (SOCS1). Indeed, SOCS levels were found to be downregulated in HCMV-infected cells transfected with a miR-182 mimic. Downstream effects of mIR-182 mimic transfection include IκB phosphorylation and subsequent NF-κB activation [74].

Zinc finger CCCH-type antiviral protein 1 is also known as ZAP, ZC3HAV1, or PARP13 [75]. There have been four isoforms of ZAP discovered to date, ZAP-S, ZAP-L, ZAPXL, and ZAPM [76]. HCMV infection was found to stimulate ZAP-S expression, and reintroduction of both ZAP-S and ZAP-L in a ZAP knockout (ZAP KO) HFF-1 cell line via lentiviral transduction reduced viral copies to wild type levels. ZAP was found to reduce mRNA levels of both UL44 (early protein) and UL83 (late protein). Consistent with this, viral protein expression overall was found to be significantly decreased. Additionally, HCMV progressed through the replication cycle more quickly in ZAP KO cells than ZAP expressing cells. Intriguingly, the researchers noted an increase in total RNA at 18 h post infection in ZAP KO cells without a corresponding increase in newly synthesized RNA, indicating that ZAP destabilizes early viral transcripts. Viral transcripts were elevated by 4 to 12-fold after 72 h in both ZAP KO and wild type cells. This was attributed to increased transcription given that ZAP wild type cells also had increased RNA levels. Later experiments determined that most genes destabilized by ZAP originated from the UL4-UL6 gene locus [75].

However, HCMV has evolved mechanisms to evade ZAP. Recent research has shown that ZAP senses CpG motifs in viral RNA through direct binding [77]. HCMV was found to suppress CpG motifs in the major immediate early (IE) genes. IE genes are expressed at the onset of herpesvirus infection and are essential for subsequent cellular events [78,79,80]. They are also thought to be involved in viral reactivation from latency as discussed elsewhere in this article. Expression of IE1, which was shown by this study to have a low CpG content, was unaffected by ZAP. Introducing mutations to increase the CpG content of IE1 resulted in ZAP mediated suppression. While infected cells expressing high levels of IE1 and ZAP were noted, cells expressing high levels of IE2 uniformly had low ZAP levels, which the authors attributed to ZAP-mediated blockage of the viral replication cycle or the possibility that ZAP expression is reduced later in the viral cycle. Transcripts expressed later in the viral replication cycle did not display the same levels of CpG suppression [81].

ISG15 is an interferon stimulated gene that belongs to the ubiquitin family of proteins [82]. ISG15 is involved in the process of ISGylation, or conjugation to its protein target. ISGylation inhibits viral growth at numerous stages of the replication cycle from cell entry to viral progeny release [83]. ISG15 has been shown to inhibit HCMV growth [84]. Infection with HCMV bearing a mutation in the UL26 gene demonstrated increased ISG15 expression as well as ISGylated proteins. Other ISGylation related genes were also upregulated, including UBA7, UBE2L6, TRIM25, and HERC5. IKK phosphorylation levels (induced by TNFα) were decreased by UL26. Lack of UL26 increased levels of another ISG, BST2. While BST2 knockdown resulted in larger wild type plaque sizes, knockdown did not affect the size of UL26 mutant plaques, indicating that UL26 is necessary to capitalize on the decreased BST2 levels. Lastly, it was shown that IKKβ knockout cells had significantly decreased ISG15 transcription levels that were comparable to mock-infected cells as well as fewer ISGylated proteins, implying that IKKβ activity is necessary for a proper ISG15 response. IKKα knockdown did not demonstrate this same inhibition [85].

Interestingly, neither ISG15 knockdown via shRNA nor ISG15 elimination via CRISPR-Cas9 affected HCMV viral replication rates. This contrasts with previous reports documenting the antiviral properties of ISG15 and ISGylation [84,86,87]. However, it is worth noting that ISG15 has been shown to attenuate the IFN response in other contexts [88,89]. The authors speculated that the lack of ISG15-mediated change in viral replication efficiency was secondary to the specific conditions of this experiment. In essence, the ISG15 knockout may have resulted in unknown changes to the typical protein ISGylation profile with subsequent unpredictable alterations in infection modulation [85].

STAT, as a key molecule in initiating IFN signaling, is targeted for downregulation by HCMV. Indeed, it was recently shown that the HCMV protein pUL145 targeted STAT. An examination of the HCVM variants AD169variantLong (AD169L) and AD169variantShort (AD169S, which lacks the ULb’ section of the genome) revealed that only the AD169L was capable of downregulating JAK2 and STAT2, although both could downregulate JAK1 and IRF9. Subsequent experiments demonstrated that engineered mutations in the UL145 gene restored STAT2 expression. Mechanistically, this is accomplished through recruitment of ubiquitin ligase complexes containing DDB1 to facilitate degradation via proteasomes. Recruitment of the ubiquitin ligase complexes was facilitated by mimicking DDB1-cullin-associated factors, a DDB1 substrate [90].

HHV-6B may use reactive oxygen species (ROS) to influence STAT phosphorylation. ROS have previously been shown to stimulate the JAK-STAT pathway [91]. A recent study by Romeo et al., demonstrated that HHV-6B upregulates ROS production. Indeed, treating infected monocytes with the ROS scavenger quercetin inhibited STAT1 and STAT3 phosphorylation. Furthermore, PD-L1 expression was noted to be increased in HHV-6B infected monocytes. Prior studies have shown that ROS can stimulate NF-κB expression in monocytes, which leads to the upregulation of PD-L1 and evolution into a monocyte secreting immunosuppressive cytokines [92]. Treatment with JAK2 inhibitor AG490 inhibited STAT1 and STAT3 phosphorylation, decreased PD-L1 expression, and decreased monocyte ROS levels. Taken together, this indicates that HHV-6B-induced STAT phosphorylation and ROS induction may play a role in PD-L1 upregulation in monocytes. As expected, STAT phosphorylation resulted in increased expression of IFN α, IL-6, IL-10, and CCL2 [93].

## 3. Immune Cell Responses

The immune system has numerous effector cells. Key cell types include but are not limited to natural killer cells, T cells, and dendritic cells [94]. These cells have a variety of immune functions ranging from cytokine secretion to antigen presentation and cytotoxic effect [95,96]. Betaherpesviruses must successfully contend with each of them in order to successfully establish and maintain an infection.

### 3.1. Natural Killer Cells

NK cells are innate lymphoid cells important in cell-mediated immunity. Effector functions include perforin and granzyme release to kill target cells as well as secretion of IFNγ, TNF, granulocyte-macrophage colony-stimulating factor (GM-CSF), CCL3, and CCL4 [96]. Their clinical significance is highlighted by patients with NK cell deficiency, who commonly develop cancers and severe, recurrent viral infections, particularly herpesvirus infections [97,98,99].

NKG2D is a key NK cell receptor that recognizes a variety of ligands encoded by MHC class I polypeptide-related sequence (MIC) A, MICB, and RAET1 gene loci. These ligands are not typically expressed by healthy cells; instead, they are induced by cellular stresses such as hyperproliferation, transformation, and infection [100].

UL148a is a HCMV protein that has recently been shown to target MICA for lysosomal degradation. Two MICA alleles were examined, a full-length MICA*004 and a truncated MICA*008. MICA*004 allele expression was partially diminished when HFF cells were infected with HCMV bearing a mutation in the UL148a gene as compared to the reduction observed in the control. It is important to note that UL148a expression alone did not diminish MICA*004 expression, which implies that other molecules are required to downregulate MICA. Levels of MICA*008 remained stable, indicating that UL148a did not target this particular MICA allele [101].

UL147a, however, does target MICA*008. In control cells, MICA*008 predominately localized to the cell membrane with some remaining in the ER. UL147a expression significantly reduced cell surface MICA*008, which was accomplished by targeting MICA*008 to lysosomes for degradation prior to leaving the ER. Specifically, MICA*008′s transmembrane domain is required for lysosomal targeting. To prove this, MICB, which is not normally targeted by UL147a, was mutated to express MICA*008′s transmembrane domain. The mutant’s surface expression was significantly decreased compared to wild type MICB. In addition, MRC-5 human lung fibroblasts were targeted for killing by NK cells when expressing a mutation in the UL147a gene [102].

Intriguingly, HCMV produces factors that augment NK cell toxicity. One such factor is cmvIL10, a human IL10 homolog that can bind to human IL10 receptors (hILR) [103]. IL10 limits inflammation in the context of a normal immune response [104], making it an attractive target for HCMV upregulation. However, cmvIL10 actually stimulates NK cell toxicity through binding to hILRs. This increase in cytotoxicity mirrors NK cell responses in the presence of human IL10. Antibodies against cmvIL10 and hIL10R reduced NK-mediated toxicity in a dose dependent fashion [105].

Like HCMV, HHV-6B has developed means by which to target NKG2D ligands. Three NKG2D ligands, MICB, ULBP1, and ULBP3, were shown to be downregulated during HHV-6B infection of SupT1, a T cell lymphoma line, while MICA and ULBP2 were upregulated. Notably, this upregulation was absent when tested in infected cord blood mononuclear cells, a primary cell. Downregulation of MICB, ULBP1, and ULBP3 was still observed. However, mRNA levels did not parallel protein levels; in fact, mRNA levels for all three NKG2D ligands were increased, indicating that repression occurs post-transcriptionally. Proteasomal inhibition rescued MICB, ULBP1, and ULBP3 expression. Interestingly, infected SupT1 cells treated with the translation inhibitor cycloheximide affected ULBP3 expression differently from MICB and ULBP1. While all ligand expression was restored when cycloheximide was given 3 and 6 hours (h) post infection, there was no difference in ULBP3 downregulation between infected and control cells at 12 h after cycloheximide treatment. This indicates that at least two viral proteins are involved in the downregulation of NKG2D receptors. Impressively, there was no difference in NK cell degranulation between cells treated with a blocking antibody against NKG2D and HHV-6B-infected cells 22 h after infection [106]. 

Targeting NK cell ligands does not represent the only means by which HHV-6A and HHV-6B can alter NK cell function; they have also been shown to infect NK cells directly, allowing them to influence NK cell miRNA and transcription factor expression. A microarray analysis examining the expression of 84 miRNAs showed that 23 were significantly affected by HHV-6A and/or HHV-6B infection. Of those, 13 miRNAs demonstrated at least a 4-fold change in expression. While some targets were conserved between the two virus species (miR-301a and miR-548e were both upregulated one day after infection, for example), other alterations in miRNA were virus specific (miR-15a and miR-21 were only upregulated by HHV-6A three days post infection, miR-590 was only downregulated by HHV-6B two and three days post infection, etc.). In addition to the above examples, the miRNAs miR-155 and miR-181, which have been implicated in NK cell cytotoxicity and maturation, respectively, were significantly downregulated. This contrasts with the effector function miRNAs miR-146 and miR-223, which were significantly upregulated in both HHV-6A and HHV-6B infection [24].

Rizzo et al., also ran a microarray to analyze the effects of HHV-6A and HHV-6B infection on transcription factor expression in infected NK cells. Results showed that infection altered expression of more than 30 transcription factors. Intriguingly, HHV-6A primarily downmodulated transcription factors early in the infection while HHV-6B upregulated transcription factor expression shortly after infection. Both viruses upregulated transcription factors by 6 days post-infection. As with the miRNAs, some transcription factors were similarly targeted by both viral species while others were species specific [24].

Killer cell immunoglobulin-like receptors (KIRs) are another class of NK cell receptors that can either inhibit or activate NK cells. Inhibitory KIRs are important in preventing NK cell-mediated destruction of healthy cells while activating KIRs stimulate intracellular signaling leading to NK cell activation [107]. HHV-6A and HHV-6B have been associated with the KIR2DL2 haplotype. For example, a recent study examined differences in NK cell activation in the context of systemic sclerosis and HHV-6 infection. Those with systemic sclerosis were more likely to have HHV-6A or HHV-6B and had higher viral titers than the control group. While they did not find any significant differences in the frequency of KIR2DL2- and KIR2DL3-expressing cells in patients with systemic sclerosis compared to the controls, those displaying the greatest degree of NK cell inhibition were noted to express the KIR2DL2 haplotype [108].

To date, there has been limited research examining HHV-7 in the context of NK cell evasion. However, U21 has been identified as a key mediator of NK cell evasion during HHV-7 infection. U21 significantly decreases surface expression of class I MHCs by rerouting them to lysosomes, which is consistent with previous reports [109,110]. In addition, HHV-7 infection was shown to downregulate surface expression of MICA and MICB as well as ULBP1 and ULBP3, although to a much lesser extent. Subsequent analysis showed that U21 altered the cellular distribution of MICA, MICB, and ULBP1, but not ULBP3. MICA and MICB molecules were almost eliminated, while ULBP1 seemed to gather in a punctate compartment reminiscent of class I MHC molecules following U21-mediated relocalization. ULBP1 was found to colocalize with the lysosomal protein LAMP2. Indeed, further experimentation demonstrated that U21 stimulated ULBP1 degradation in lysosomes. MICA was not examined due to the similarity between MICA and MICB and better visualization of MICB. Inhibition of lysosomal proteases increased MICB concentration, indicating that at least some MICB is targeted for lysosomal degradation. However, this was not significantly different than increases in MICB stability noted in cells not expressing U21. Instead, U21 decreased the steady-state MICB concentration. Additionally, the authors found that MICB migrated slightly faster in pulse-chase experiments. Digestion with PNGase:F did not impact protein mobility, indicating that the increase in migration speed was likely due to post-translational modifications rather than changes in the core protein. These alterations decreased NK cell killing of infected cells relative to the control. Notably, blocking MICA and MICB decreased NK cell killing of infected cells to the level seen in U21-expressing cells, indicating that ULBP1 plays a relatively minor role in this process. Overexpression of ULBP1 did increase NK cell killing of target cells, which is consistent with the hypothesis that ULBP1 may not be expressed in sufficient quantities to stimulate NK cytotoxicity [111].

### 3.2. T Cells

Two primary divisions of T cells exist: CD4+ T cells and CD8+ T cells. There are several classifications of CD4+ T cells, including T-helper (TH) 1, TH2, TH9, TH17, and follicular helper T cells. These cells secrete specific cytokines to modulate the immune response. Their immunomodulatory functions encompass both the activation of innate immune cells and the repression of immune responses [112]. While the majority of CD4+ T cells die following an infection, a subset survives as memory T cells. These cells can then be reactivated following re-exposure to an antigen, paving the way for a more robust immune response [113]. CD4+ T cell activation is characterized by their interaction with major histocompatibility complex (MHC) class II molecules found on antigen presenting cells (APCs) [112]. Using mass spectrometry, Becerra-Artilles et al., recently identified six HHV-6B proteins that, once processed, yielded 25 peptides that were recognizable by MHC class II receptors and stimulated a CD4+ T cell response. These six proteins were U11, U39, U48, U56, U63, and U85, which are a tegument protein, glycoprotein B, glycoprotein H, a capsid component, a hypothetical protein, and a CD200 homolog, respectively. Responses were primarily characterized by IFN-γ production, with fewer cells secreting TNFα and IL-2. A subset of cells expressed CD107a, a degranulation marker. Many of the responding cells produced more than one type of response. In vitro cytotoxicity assays demonstrated targeted killing of cells containing viral peptides [114].

Both class I and class II MHC complexes are targeted by betaherpesvirus immunoevasive strategies [115,116]. Recent research has uncovered a novel mechanism by which HCMV downregulates MHC class II molecules. Infection of Kasumi-3 cells, a myeloid progenitor line, resulted in significantly lower levels of both surface and total human leukocyte antigen DR (HLA-DR), an MHC class II complex [117]. Subsequent experiments showed that HLA-DRα transcript levels were within normal limits at 24 h post infection but significantly decreased at 72 h post-infection [117]. MHC class II transactivator (CIITA) is essential in appropriate expression of MHC class II complexes [118]. CIITA transcript levels paralleled HLA-DRα levels, implying that CIITA transcript repression is a mechanism by which HCMV downregulates MHC class II expression [117]. CIITA transcription itself is controlled by several promoters (pI-pIV), although the function of pII has not been closely examined [118]. While the authors were able to determine that CIITA downregulation occurs when immediate early or early genes target promoter III transcription or promoter III transcripts, the precise mechanism has not yet been worked out [117].

The paucity of HHV-6A and HHV-6B-specific T cells has been cited as one of the prevailing difficulties in studying the T cell response to these viruses [119], a finding confirmed in a recent study by Fastenackels et al., assessing the T cell response to HHV-6 antigens. The study in question selected the HHV-6A and HHV-6B antigens U54, a positional homologue to HCMV pp65, and U90, a positional homologue to HCMV IE1. When challenged with lysates containing these HHV-6 antigens, the CD4+ HHV-6B specific T cell response as measured by percent of cells secreting IFNγ was driven by the U90 protein product over the U54 protein product. This bias towards U90 did not hold for TNFα secretion. The authors next allowed the cells to sit in lysates containing U90 antigen, U54 antigen, or HCMV antigen. Interestingly, T cells in the HCMV lysate exhibited a greater proportion of intermediate/late effector memory T cells than those left in lysates containing U90 and U54, which contained a greater proportion of central memory and early effector memory T cells. CD8+ T cells targeting HHV-6 exhibited the same elevated frequency of less differentiated central memory and decreased frequency of intermediate/late effector memory T cells. This is consistent with HHV-6-specific T cells being less differentiated than their HCMV counterparts. HCMV-specific and HHV-6A and HHV-6B-specific T cells also differed in the quantity of regulatory T cells (Tregs) generated. HHV-6A and HHV-6B-specific Tregs comprised a greater percentage of HHV-6A and HHV-6B-specific T cells than HCMV-specific T cells, a trend that was also seen in the relative frequencies of effector Tregs [120].

CD8+ T cells are capable of both target cell lysis and secreting cytokines such as IFNγ and TNFα. Like CD4+ T cells, a portion of CD8+ T cells survive as memory T cells following infection. Unlike CD4+ T cells, CD8+ T cells are activated by peptides presented on MHC class I molecules [121]. A recent advance in the interaction between MHC class I molecules and HCMV came about as an unexpected discovery during research to create a cytomegalovirus-based vaccine for glioblastoma. It was noted that E6-specific T cells were not activated by glioblastoma cells infected with HCMV-based vaccines including the human papillomavirus proteins E6 and E7 [122]. The proteins US2, US3, US6, and US11, all of which are known to downregulate MHC class I presentation [123,124,125], had been removed. U251 cells were made to express E6 and E7. Those infected by the HCMV vector lacking US2, US3, US6, and US11 stimulated E6-specific T cell reporter cells less than 50% as effectively as uninfected cells. The mechanism of this unknown block has not yet been established and represents an avenue of further research [122].

Evasion of these cellular responses during active infection can lead to symptomatic disease. Working with a murine cytomegalovirus and mouse model that included an MHC class I mismatch between the donor and recipient of a hematopoietic cell transplant, Holtappels et al., demonstrated that lethal infections in the setting of graft v. host (GvH) reaction occurred due to poor reconstitution of virus-specific CD8+ T cells. This led to an inability to appropriately recruit CD8+ T cells to form nodular inflammatory foci. Deaths only occurred in mice suffering from GvH reaction and not host v. graft (HvG) reactions. Notably, viral loads were higher in the spleen, liver, and lungs but not the salivary glands of mice with GvH reactions. CD8+ T cells have previously been shown to be essential in preventing post-transplant multiple organ failure secondary to CMV [126]. Nodular infective foci are conglomerations of virus-specific T cells that prevent viral spread [127]. Liver histology from mice suffering from HvG reactions demonstrated nodular infective foci while T cell infiltrates in the liver of mice suffering from GvH reactions were randomly distributed, indicating that the infiltrative T cells were not protective against HCMV. Further analysis showed that existing T cells in GvH reaction mice were unable to recognize MHC class I peptide concentrations lower than 10^−9^, which the authors estimated was the minimum concentration at which T cells must recognize viral antigens to detect infected cells [128].

The authors next examined whether mice with GvH reactions would survive if infected with a cytomegalovirus strain with mutations in the immunoevasive genes governing antigen presentation. Notably, mice infected with the recombinant virus all survived. Pre-treating cells with IFNγ also increased the number of cells capable of responding to both wild type and mutant cytomegalovirus, indicating that IFNγ can improve antigen presentation [128]. 

Martin et al., recently examined antigen presentation by the MHC complex HLA-B*08:01 in HHV-6 infection. HLA-B*08:01 was selected because of its frequency in the human population, the strength of response, and the relatively conserved consensus motif. Screening detected 146 octameric and 153 nonameric peptides that conformed to this motif. Donor T cells expressing HLA-B*08:01 were stimulated with octamer and nonamer peptide mixes followed by weekly restimulation by B cells containing these mixes. Cloned T cells were able to recognize 25 HHV-6B peptides originating from 19 distinct open reading frames or proteins. Of the 17 peptide-specific T cell clone lines examined, 13 were able to recognize CD4+ T cells infected with either HHV-6A or HHV-6B, and 16 of the 25 peptides were shown to be epitopes presented by CD4+ cells infected with either HHV-6A or HHV-6B. The T cells did not recognize four peptides and five peptides went untested due to insufficient T cell survival [129].

HHV-6A has been shown to modulate expression of both HLA class I and class II molecules in infected mesothelial cells. HLA-G, a nonclassical class I molecule, was the exception in that it was induced. While HLA class I molecule expression was depressed, HLA class II molecule expression was induced. The authors speculated that HLA class II expression may have an antigen presentation-like function because mesothelial cells do not typically express these molecules. In fact, when CD4+ T cells were stimulated with the anti-CD3 antibody OKT3 and cultured with mesothelial cells, they observed a five-fold increase in T cell proliferation relative to mock-infected cells [130].

T lymphocytes infected with HHV-6A demonstrated marked alterations in their miRNA expression. Several miRNAs, including miR-16 _1, miR-34a, miR-130a, miR-202, miR-301b, miR-302c, and miR-449b, were significantly upregulated. T cell infection with HHV-6B and HHV-7 did not modulate miRNA expression to nearly the same degree [131].

As was previously mentioned, it has long been known that the HHV-7 glycoprotein U21 shunts MHC class I complexes to the lysosome [110]. However, the mechanism by which this is accomplished has not yet been fully explored. Dirk et al., utilized the retention using selective hooks (RUSH) method (described in [132]) to observe the MHC complex HLA-A2 and U21 trafficking through the cell. While HLA-A2 complexes tagged with RUSH typically localize to the cell membrane after leaving the Golgi in tubules, RUSH-tagged HLA-A2 complexes were absent from tubules when co-expressed with RUSH-tagged U21. However, HLA-A2 was present in all vesicles containing RUSH-tagged U21 [133].

It had previously been established that U21 oligomerizes with MHC class I complexes [134] and that oligomerized Golgi proteins are targeted to lysosomes for degradation [135]. This led to the hypothesis that the U21/MHC class I oligomers exit the Golgi apparatus in lysosome-bound vesicles [133]. Specifically, the authors postulated that the U21/MHC class I oligomers exited the Golgi apparatus in quality control carriers. These recently discovered carriers transport unfolded proteins, protein aggregates, and oligomers resembling protein aggregates [136].

### 3.3. Dendritic Cells

Dendritic cells (DCs) serve as a bridge between the innate and adaptive immune response [137]. They play an integral role in antigen presentation and T cell stimulation, making them indispensable for the induction of adaptive immunity, and they are also thought to be essential in establishing immunotolerance [95,137]. As such, they represent important targets for immunomodulation.

CD83 acts as a marker for dendritic cells [138] that multiple viruses target for immunomodulation, including HCMV [139]. Specifically, HCMV downregulates CD83 expression via the proteasome. Decreased expression levels were noted both intracellularly and at the cell surface. HCMV-infected mature DCs treated with proteasome inhibitors had unchanged CD83 levels while non-treated HCMV-infected cells exhibited a significant decrease in surface CD83 levels within 12 h of infection, indicating that HCMV targets CD83 for proteasomal degradation. Subsequent experiments demonstrated that IE2 was sufficient to stimulate CD83 downregulation. In contrast to previous reports [140], no significant difference in the levels of soluble CD83 in the supernatant of HCMV-infected and mock-infected cells was detected [141].

It was recently proposed that HCMV impedes the immune response by altering DC migration. While transwell migration of cells to the chemokine CXCL12 was unaffected, migration towards the chemokine CCL19 and migration in the absence of chemokines were both inhibited. Interestingly, expression of the surface receptor CCR7, which binds CCL19, was not affected until 24 h, and only modestly downregulated afterwards. CXCR4, which binds CXCL12, was actually upregulated during HCMV infection. While chemokine receptor modulation did not appear to play a role in the impaired chemotaxis, the authors noted that HCMV-infected cells adhered to fibronectin and ICAM more strongly than controls. Subsequent experiments demonstrated integrin activity in infected cells due to HCMV-mediated inhibition of the β2-integrin negative regulator cytohesin-1 interacting protein (CYTIP). CYTIP levels were mostly restored after administration of MG-132, a proteasome inhibitor. In summary, HCMV targets CYTIP for proteasomal degradation, which leads to increased β2-integrin activity and decreased chemotaxis in response to CCL19. Interestingly, CCL19 is important in the chemotaxis of CCR7-bearing cells to secondary lymphoid organs [142] while CXCL12 is expressed in the bone marrow [143]. This led the authors to postulate that disruption of CCR7/CCL19 signaling and retention of CXCL12 signaling reroutes DC migration from secondary lymphoid organs to the bone marrow [144].

HHV-6A infection of DCs can cause DC cell death [145]. High mobility group box 1 (HMGB-1) is a protein located in the nucleus that acts as an alarmin and is capable of stimulating the innate immune system through either active release or passive release following cell death [146]. Levels of HMGB-1 were elevated in the supernatant following HHV6-A infection of dendritic cells, which the authors postulated may increase the risk of developing inflammatory diseases such as multiple sclerosis. In addition, infection altered the cytokine profile. While TH1 cytokine expression was unaffected, levels of cytokines produced by TH2 cells were significantly elevated relative to the control [145].

## 4. Autophagy and Apoptosis

**Figure 4:**
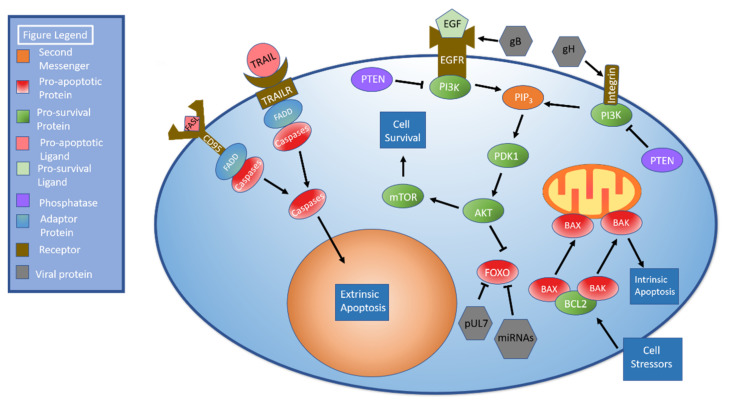
AKT and Apoptosis. There are two apoptotic pathways: extrinsic and intrinsic. The extrinsic pathway begins when ligands such as FAS ligand (FASL) or TRAIL bind their corresponding cellular receptors. The adaptor protein FADD is recruited to the receptor. FADD recruits procaspases to create the death-inducing signaling complex (DISC). Once DISC has formed, it facilitates autoproteolytic cleavage and caspase activation. Activated caspases cleave target molecules and stimulate apoptosis. The intrinsic pathway is initiated when the cell is exposed to stressors. The pro-apoptotic proteins BAK and BAX dissociate from BCL2 and disrupt the mitochondrial membrane, resulting in release of mitochondrial contents and apoptosis. The AKT pathway, which can be initiated through either EGFR or integrin stimulation, begins with receptor-mediated activation of PI3K. PI3K activation results in the formation of PIP_3_, which activates PDK1, which in turn activates AKT. AKT activates mTOR, which promotes cell survival. The human cytomegalovirus (HCMV) protein pUL7 and HCMV miRNAs inhibit FOXO. HCMV gB interacts with the EGFR while gH interacts with integrins to stimulate AKT.

Apoptosis can be triggered by both intra- and extracellular signals. Cell death mediated by external signaling, termed extrinsic apoptosis, is characterized by the binding of several different molecules including FAS/CD95L and TNF related apoptosis inducing ligand (TRAIL). These bind to the receptors CD95 and TRAIL receptors 1 and 2, respectively [147]. When stimulated, these molecules recruit FADD, which recruits procaspases to create a death-inducing signaling complex (DISC). Once created, DISC facilitates autoproteolytic cleavage and activation of caspases. Activated caspases cleave molecules such as lamin A, poly (ADP-ribose) polymerase (PARP), and inhibitor of caspase-activated DNase (ICAD) to stimulate apoptosis [148]. The intrinsic pathway is triggered following exposure to stressors such as DNA damage or nutrient deprivation. These stressors cause the proteins BAX and BAK to dissociate from BCL2 and damage the mitochondrial membrane, leading to perforation of the mitochondrial membrane and subsequent release of mitochondrial contents [149]. Figure 4 details the apoptotic pathways.

Viruses are obligate intracellular parasites, meaning they require living cells for their survival. Thus, it stands to reason that viruses have strategies to subvert the apoptotic pathway. In fact, Arcangeletti et al., determined that both HCMV and HHV-6A significantly altered the expression levels of numerous apoptosis-associated genes, with HCMV having a greater influence on apoptotic genes than HHV-6A [150].

Monocytes are essential to HCMV persistence and dissemination [151]. However, monocytes have a relatively short lifespan of only a few days [152]. HCMV has been shown to induce monocyte differentiation into longer-lived macrophages that support HCMV replication [151]. Recent research has shed light on the effect of modulating the AKT signaling pathway on monocyte survival [153]. In brief, PI3K activation leads to the formation of PIP_3_, which activates 3-phosphoinositide-dependent kinase 1 (PDK1). PDK1 phosphorylates and activates AKT. AKT stimulates mTOR signaling, which leads to activation of anabolic pathways as shown in Figure 4 [154]. Of note, both EGFR [155] and integrins [156] have been shown to stimulate AKT signaling.

Cojohari et al., first described how HCMV induces AKT within 15 min of infection to allow monocyte survival beyond 48 h, which is accomplished by altering expression of the AKT modulating factors PI3K, PTEN, and SHIP1. Moreover, HCMV activation of the AKT pathway was stronger than that induced by macrophage colony stimulating factor (M-CSF) under physiologic conditions [157]. It was later shown by Peppenelli et al., that the viral glycoproteins gB and gH interacted with epidermal growth factor receptor (EGFR) and αvβ3 integrin, respectively, to stimulate the AKT pathway. These interactions increased myeloid leukemia sequence 1 (Mcl-1) and heat shock protein 27 (HSP27) to deter apoptosis within monocytes [158]. Mahmud et al. further expanded on this research, determining that gB may have a more significant effect on AKT signaling given that monocytes treated with gB inhibitors alone had lower survivability than those treated with gH alone. While soluble gB and gH could stimulate monocyte survival, monocytes did not survive as well as when infected by HCMV. This may indicate that co-stimulation by gB and gH is necessary to fully potentiate AKT stimulation. Additionally, they showed that gB binds EGFR and that gH binds integrin β1 but not β3 in monocytes. This finding contrasts with the above findings of Peppenelli et al., who found that gH binds αvβ3 integrin. One potential explanation for this discrepancy is that Peppenelli studied fibroblasts while Mahmoud studied monocytes. Interestingly, treatment with either an integrin or EGFR inhibitor blocked signaling through both integrin β1 and EGFR, implying some degree of signal crosstalk. Elimination of this crosstalk blocked HCMV-mediated AKT signaling. EGFR inhibition resulted in abrogated upregulation of MCL-1 and HSP27, which are anti-apoptotic proteins generated by the AKT signaling cascade. The simultaneous activation of EGFR and integrin β1 results in noncanonical activation of AKT signaling. Lastly, inhibition of SHIP was shown to downregulate the pro-survival proteins Mcl-1 and HSP27 [159]. This contrasts with its normal inhibitory function [154]; however, aberrant SHIP function has been previously demonstrated in cancer cells [160].

FOXO is a pro-apoptotic protein family targeted by AKT to promote cell survival [161] and represents another point at which HCMV can regulate apoptosis. HCMV protein pUL7 stimulates the phosphorylation, translocation to the cytoplasm, and inactivation of FOXO3a through the MAPK pathway. Telomerized human fibroblast cells treated with pUL7 had significantly lower levels of FOXO3a downstream targets BCL2L11 (BIM) and CDKN1B (p27). mRNA expression and protein levels of FOXO3a were unchanged, indicating that regulation occurred via phosphorylation rather than inhibition or degradation, but pUL7 did decrease mRNA levels of the pro-apoptotic protein BCL2L11 [162].

miRNAs represent another means of FOXO3a targeting and subsequent anti-apoptotic regulation. Transfection of HEK293T cells with HCMV miR-US5-1 and miR-UL112-3p was found to target the 3′ UTR of FOXO3a, which caused FOXO3a transcript and protein levels to decrease [162].

Autophagy is essential to cellular function. Not only does this process dispose of worn cellular components, but their breakdown is an important source of energy and materials to synthesize new components [163]. Autophagy is also integral to the normal immune response with functions including but not limited to antigen presentation and pathogen destruction [164].

Viruses are known to manipulate the autophagic process for their survival and benefit [164]. Romeo et al., noted autophagosomes, which are involved in the autophagic process, in both infected and nearby uninfected cells as well as an increase in lipidated LC3II, a marker of autophagic flux, in HSB-2 cells after infection with HHV-6A. By comparison, HHV-6B was found to block autophagy in infected Molt-3 cells. Autophagy blockage was confirmed by the relative paucity of autophagolysosomes in both infected and nearby uninfected cells as well as the accumulation of p62, a protein that is normally degraded by autophagy. The two were found to differentially regulate the unfolded protein response [165], which is associated with endoplasmic reticulum stress. Endoplasmic reticulum stress triggers autophagy, which can help restore the endoplasmic reticulum to its baseline state. If the stressor is too severe, apoptosis ensues [166]. While HHV-6A increased expression of immunoglobulin heavy chain binding protein (BiP), an anti-apoptotic protein, HHV-6B upregulated C/EBP homologous protein (CHOP), a pro-apoptotic protein. This was accomplished by modulating expression levels of the upstream factors inositol requiring enzyme 1 alpha (IRE1α), activating transcription factor 4 (ATF4), and activating transcription factor 6 (ATF6) [165].

**Figure 5:**
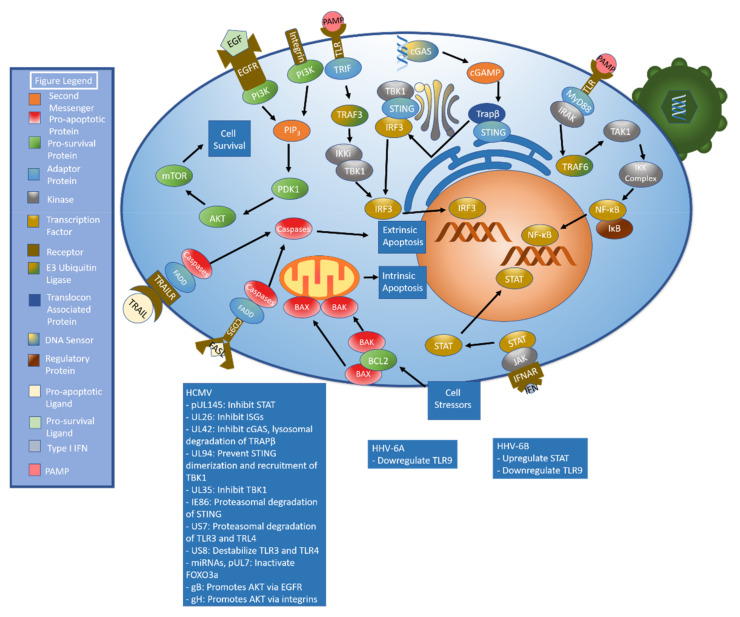
Summary of Key Pathways and Associated Immunoevasive Methods. The immune response to betaherpesviruses begins with the detection of pathogen-associated molecular patterns (PAMPs) by pattern recognition receptors (PRRs), including cGAS and toll-like receptors (TLRs). The cGAS/cGAMP/STING signaling axis results in the production of interferon (IFN) while TLRs can generate both IFN and other defensive compounds. IFN results in STAT activation, which stimulates production of interferon-stimulated genes (ISGs). Betaherpesvirus manipulation of cell survival is an integral element in their success as pathogens. Apoptosis may be stimulated by either intrinsic (BAK/BAX mediated mitochondrial perforation) or extrinsic (FAS ligand/CD95 binding, TRAIL/TRAILR binding) signals. The PI3K/AKT/mTOR signaling pathway represents a major pro-survival cascade. Immunoevasive methods are listed below.

Autophagy dysregulation has also been shown to play a role in inhibiting dendritic cell formation and monocyte survival in HHV-6B infection. HHV-6B extracted from patients with exanthem subitum was able to inhibit monocyte differentiation into dendritic cells and decrease overall monocyte survival. The addition of sodium 4-phenylbutyrate, which reduces endoplasmic reticulum stress by preventing protein aggregation and acting as chaperone to stabilize folded proteins, attenuated the HHV-6B-mediated inhibition of monocyte differentiation and improved monocyte survival [167]. A general summary of betaherpesvirus immunoevasive strategies can be found in the table below (Table 1). A graphical summary of pathways discussed above with a listing of key immunoevasive strategies can also be found below (Figure 5).

## 5. Latency and Reactivation

Upon infection, betaherpesviruses establish lifelong latency in the human host. The virus can subsequently be reactivated under certain conditions and enter a lytic phase. Latency and reactivation play an important role in betaherpesviruses’ evasion of the human immune system, and there are many facets to the control of these processes [4].

### 5.1. Viral Tropism

Before establishing latency, the virus must first gain entry into and infect a cell. As with all viruses, betaherpesviruses display cellular tropism and can only infect certain cell types [4]. A variety of factors influence the tropism of betaherpesviruses. Betaherpesvirus tropism was discussed briefly in the introduction of this review, and recent studies examining this topic will be explored here.

Recent research has indicated that HCMV entry into CD34+ human progenitor cells (HPCs) is mediated in part by the activation of epidermal growth factor receptor (EGFR) signaling. EGFR signaling is also suggested to play a role in the establishment of latent HCMV infection in CD34+ HPCs by regulating several key early steps. Inhibition of EGFR signaling with AG1478, an EGFR kinase (EGFRK) inhibitor, resulted in decreased latency-associated UL138 mRNA and increased lytic IE1/IE2 mRNA transcripts. Treatment of HCMV-infected CD34+ cells with AG1478 suppressed mRNA expression of the cellular hematopoietic cytokine interleukin 12 (IL-12), while mock-infected cells were unaffected. Collectively, the results indicate that EGFR signaling contributes to the determination of HCMV tropism by affecting hematopoietic potential [168]. However, a study by Buehler et al., discussed later in regard to the maintenance of latency, found slightly different results relating to HCMV and EGFR signaling. The authors suggest that EGFR signaling is downregulated by HCMV in the early stages of infection and that EGFR signaling promotes latency later in infection [169].

Shnayder et al., recently presented a new understanding of latent HCMV infection, contradicting previous views that suggested a specific latency-associated viral program of gene expression. Through analysis of a large transcriptome RNA sequencing (RNA-seq) atlas as well as single-cell RNA-seq (scRNA-seq) of latently-infected CD14+ monocytes and CD34+ HPCs, this study found that the gene expression profile during HCMV latency may actually resemble that of the late lytic phase of the virus. They described a quantitative rather than qualitative change in gene expression, with simply a lower level of gene expression during latency. This challenges the idea that there is a latency-specific program of genetic expression for HCMV [170].

### 5.2. Establishment of Latency

Establishment of latency is another mechanism by which betaherpesviruses evade the immune system. Chromosomal integration is a highly regulated event that is important to the establishment of betaherpesvirus latency [4]. HHV-6A and HHV-6B are known to integrate their genomes into the telomeres of human chromosomes in several different cell types, including germinal cells [171]. The HHV-6A genome in particular is transcriptionally silent after chromosomal integration. In human 293T cells infected with HHV-6A, viral transcription is progressively silenced and is nearly undetectable by seven days post infection. The chromosomally integrated HHV-6A genome exists as condensed heterochromatin [172] and appears to reside in a compartment similar to a topologically associated domain (TAD), which is characterized by a high number of local intrachromosomal interactions [171]. A recent study examined higher-order chromatin interactions involving chromosomally integrated HHV-6A using circular chromosome conformation capture assays (4C-seq) specific to HHV-6A. The 4C-seq assays found that most virus-host chromatin interactions were with heterochromatin, indicating that integrated HHV-6A is able to interact with repressed chromatin. These interactions may play a role in silencing viral gene expression in cells with chromosomally integrated HHV-6A [171].

As mentioned earlier, HHV-6A/B can integrate into germinal cells [171,173], allowing HHV-6A/B to be transferred vertically. This vertical DNA transfer results in a phenomenon known as inherited chromosomally-integrated HHV-6 (iciHHV-6), where every nucleated cell contains the integrated HHV-6 genome [173]. Previous studies have shown that telomeric repeats of HHV-6A are vital for integration [174], while the putative viral integrase U94 is dispensable since efficient integration of HHV-6 is possible in the absence of U94 [175]. Further research in this area has found that the potential viral recombination proteins U41 and U70 are also nonessential for telomere integration of HHV-6A since cells expressing shRNAs against U41 and U70 still demonstrated successful telomere integration [173]. When the cellular recombinase Rad51 was inhibited using the small molecule inhibitor RI-1, HHV-6A still integrated efficiently, indicating that Rad51 is also dispensable [173].

However, recent research has elucidated some aspects of HHV-6 integration. HHV-6B infection of Molt-3 T cells revealed CpG hypomethylation close to the telomeres of chromosomes. Chromosome 17p13.3 was most strongly affected, and the genes in this region were furthermore found to have higher levels of gene expression. HHV-6B also modulates the expression of ten-eleven translocation (TET) enzymes, which play a role in active demethylation. TET2 expression in particular was found to be significantly upregulated by HHV-6B. Given 17p is known to be an integration site for HHV-6A/B, this observed hypomethylation may play a role in viral integration by making the host DNA more accessible [176].

Shelterin proteins, which protect host telomeres by preventing the activation of DNA damage recognition pathways, have been found to play a role in HHV-6A/B integration. The ends of HHV-6A/B virus genomes contain many tandem repeats similar to those found in telomeres. In fact, HHV-6A infection resulted in a 2.5 to 2.9-fold increase in the number of telomeric repeats relative to uninfected cells. These repeats were determined to be of viral origin. Cellular shelterin protein TRF2, which normally binds host telomeres, was shown to bind viral telomeric repeats as well during HHV-6A/B infection. Viral immediate early 2 (IE2) protein colocalized with TRF1, TRF2, and telomeres in the context of HHV-6A infection. TRF2 knockdown resulted in reduced IE2 localization at cellular telomeres and also significantly reduced HHV-6A/B integration frequency. These results underscore the importance of this protein in HHV-6A/B integration [177].

Another protein implicated in HHV-6B integration is the cellular promyelocytic leukemia protein (PML). PML forms nuclear bodies (PML-NBs), which play a role in the host antiviral defense. HHV-6B immediate early 1 (IE1) protein, which regulates early viral gene expression and is instrumental in lytic replication of the virus, colocalizes with PML independent of other viral factors. In the presence of PML-NBs, IE1 was hyperSUMOylated, likely at multiple SUMO acceptor sites (referred to as “multiSUMOylation”). This multiSUMOylation was dependent on the presence of the putative ^775^VIV^777^ SUMO Interacting Motif (SIM) site as well as the K802 SUMO acceptor site on IE1. The ^775^VIV^777^ SIM on IE1 is also important for the oligomerization of IE1 with PML-NBs. HHV-6B integration frequency was significantly reduced in PML knockout cells, while restoration of PML rescued the integration frequency, indicating that PML is required for HHV-6B integration. Finally, IE1 localization at telomeres in the context of HHV-6B infection was dependent on the presence of PML. These results illustrate the importance of PML in HHV-6B integration [178].

In summary, while progress has been made in understanding how betaherpesviruses integrate their genomes into host chromosomes, further investigation is needed to better elucidate the mechanisms by which these viruses establish a latent infection.

### 5.3. Maintenance of Latency

Maintenance of viral latency is also highly regulated in betaherpesviruses. As previously mentioned in the discussion of viral tropism, the EGFR pathway plays a role in HCMV replication. In contrast to the earlier study, which found that the activation of EGFR signaling mediates HCMV entry into CD34+ HPCs as well as the establishment of latency [168], Buehler et al., assert that HCMV actually downregulates EGFR and decreases EGFR activation early in infection. In the context of latency, HCMV infection in CD34+ HPCs sustained a low basal level of EGFR activity. Inhibition of signaling downstream of EGFR increased viral reactivation from CD34+ HPCs latently infected with HCMV, leading the authors to examine EGFR signaling in relation to latency. Stimulation of EGFR increased expression of the viral gene UL138, a determinant of latency, suggesting that EGFR signaling does indeed promote latent gene expression. Downstream pathways of EGFR also suppress viral replication in order to maintain latency. One downstream effector of the EGFR signaling pathway is the transcription factor early growth response gene 1 (EGR1), which is important in maintaining stem cell quiescence. The authors discovered that EGR1 binds the HCMV genome upstream of UL138 and promotes UL138 expression. Inhibiting EGR1 binding through mutation blocked UL138 expression and prevented the establishment of latency, signifying the importance of EGR1 to latency [169].

HCMV encodes a variety of microRNAs (miRNAs), which regulate both cellular and viral gene expression and often play a role in latency and reactivation. Several of these miRNAs have recently been shown to affect TGF-β signaling. Studies found that in latently-infected CD34+ HPCs, HCMV induced secretion of the cytokine TGF-β, which regulates hematopoietic cell differentiation and proliferation. HCMV-induced TGF-β secretion was discovered to cause myelosuppression in CD34+ HPCs since the presence of a TGF-β neutralizing antibody restored myeloid colony formation to levels observed in control cells. HCMV utilizes the latent viral gene product miR-US5-2 to mediate this increase in TGF-β by targeting NAB1, a transcriptional repressor of transcription factor EGR1. miR-US5-2 thus indirectly causes increased EGR1, which subsequently activates TGF-β expression. Studies found that miR-US5-2 was sufficient to induce both myelosuppression and TGF-β expression in CD34+ HPCs, as miR-US5-2 deletion restored both HPC proliferation and myelopoiesis and decreased TGF-β secretion [179].

Another miRNA, miR-US25-1, also promotes the maintenance of latent HCMV infection. The GTPase RhoA was determined to be a target of miR-US25-1 as expression of this miRNA in HEK293T cells and normal human dermal fibroblasts (NHDFs) reduced endogenous protein levels of RhoA by approximately 50%. When NHDFs were infected with a mutant virus lacking miR-US25-1, RhoA expression was increased relative to infection with wild type HCMV. miR-US25-1 also affected downstream RhoA signaling, further supporting this conclusion. The activation of myosin II, which is vital for cell proliferation and cytokinesis, serves as an example of impaired RhoA signaling. NHDFs expressing miR-US25-1 did not appear to undergo cytokinesis, suggesting that mitotic dysregulation is one mechanism through which this miRNA disrupts cellular proliferation. Finally, infection of CD34+ HPCs with wild type HCMV and a mutant lacking miR-US25-1 showed that miR-US25-1 increases the retention of latent viral genomes because cells infected with the mutant virus contained fewer genomes relative to those infected with wild type virus. As a whole, these results indicate that miR-US25-1 modulates RhoA signaling in order to restrict proliferation of CD34+ HPCs and increase retention of the latent viral genome, which can be lost through proliferation [180].

The HCMV gene US28, which encodes a cell surface G protein-coupled receptor (GPCR), is one of the few genes expressed during latency [181]. However, US28 is also expressed during lytic infection, when it acts as a constitutive activator of cell-signaling [182]. HCMV can establish latent infections in monocytes since they arise from myeloid lineage cells [183]. A recent study found that US28 is required to establish a latent HCMV infection in monocytes as infection with a mutant virus lacking US28 resulted in full lytic infection. In contrast to its function during lytic infection, US28 attenuates mitogen-activated protein (MAP) kinase and NF-κB signaling in latency. When treated with inhibitors of both MSK-1 and IκB kinase to block MAP kinase and NF-κB signaling, respectively, lytic infection was reduced in monocytes infected with a US28 deficient virus. This further supports that the attenuation of MAP kinase and NF-κB signaling by US28 promotes latent rather than lytic infection. Moreover, HCMV-infected monocytes treated with a US28 inverse agonist, VUF2274, also underwent lytic infection. Finally, monocytes infected with a mutant virus lacking US28, which can only undergo lytic infection, were targets for cytotoxic T lymphocytes specific to HCMV. Latently-infected cells treated with VUF2274 undergo lytic reactivation and also become susceptible to the cytotoxic T lymphocyte response, making US28 a potential therapeutic target [184]. 

A study by Crawford et al., also examined HCMV US28, this time from the perspective of protein tyrosine kinase signaling in CD34+ HPCs. US28, an HCMV chemokine receptor, acts through both ligand-dependent and independent mechanisms in CD34+ HPCs. However, Crawford et al., found that constitutive US28 signaling was required for reactivation but not for establishing or maintaining latency. This contrasts with the findings described above from Krishna et al., which suggest that US28 is required for latency in monocytes [184]. In the experiments performed by Crawford et al., CD34+ HPCs infected with wild type virus were able to reactivate while cells infected with a recombinant HCMV strain disrupting US28 protein expression were not. However, both cells infected with wild type virus and cells infected with recombinant virus were able to establish and maintain latency. Additionally, they found that absent US28 constitutive signaling did not affect latency while US28 ligand binding activity was required for latency. This last finding was true in vitro for CD34+ HPCs and in vivo for the NOD-*scid* IL2Rγc null (huNSG) humanized mouse model [185].

Another study examining the HCMV protein GPCR US28 confirmed that US28 suppresses lytic gene expression. When expressed at the time of infection, US28 represses major immediate early promoter (MIEP)-driven lytic transcription within 24 h, but US28 expression must be continuous for this effect to be present. This subsequently decreases viral production, suggesting that US28 plays a key role during latency. US28 also targets the cellular fos (c-fos) subunit of transcription factor AP-1, reducing c-fos expression and signaling. Finally, this attenuation of c-fos signaling was determined to reduce MIEP activity and subsequent infectious virus production in latently-infected Kasumi-3 cells, indicating the importance of US28 to the establishment and maintenance of latency [186].

One of the strategies that HHV-6A utilizes to maintain latency is the alteration of host cell metabolism. Experiments using the T-lymphoblastoid cell line HSB-2 infected with HHV-6A found that metabolism-related genes, particularly those for glycolysis, were upregulated. Glucose consumption, glycolysis metabolite production, lactic acid secretion, and the extracellular acidification rate (ECAR, a marker of glycolysis) were all increased, indicating that glucose metabolism is increased in HHV-6A-infected T cells. mRNA and protein expression levels of the glucose transporters Glut1 and Glut3 were also significantly increased in HSB-2 cells infected with HHV-6A. HHV-6A infection also induced the relocalization of these transporters to the cell membrane, indicating that the transporters are indeed functional. AKT-mTORC1 signaling, which regulates a variety of cellular processes including energy metabolism, was activated in infected cells, and rapamycin-induced mTORC1 inhibition resulted in obstruction of HHV-6A-induced glycolytic activation, confirming the role of AKT-mTORC1 signaling in this process. Finally, pharmaceutical inhibition of either glycolysis or of mTORC1 activity reduced viral replication, suggesting that both are vital for the efficient propagation of HHV-6A [187].

### 5.4. Viral Reactivation

Once betaherpesviruses establish a latent infection, the virus can reactivate under certain conditions. The above study by Crawford et al., which reported that the ligand binding activity of the HCMV GPCR protein US28 is required for latency in CD34+ HPCs and in the NOD-*scid* IL2Rγc null (huNSG) humanized mouse model, found that US28 is required for reactivation as well in these models. They also demonstrated that US28 promotes the differentiation of CD34+ HPCs toward the myeloid lineage, which is more favorable for reactivation. US28 thus plays a role in the regulation of both latency and reactivation [185].

Another HCMV protein, UL7, has also been found to promote differentiation. This glycoprotein binds the Fms-like tyrosine kinase 3 receptor (Flt-3R) and subsequently activates both the phosphatidylinositol 3-kinase (PI3K)/AKT and the mitogen-activated protein kinase (MAPK)/extracellular signal-regulated kinase (ERK) signaling pathways in CD34+ HPCs [188]. Flt-3R plays a crucial role in HPC differentiation [189], and accordingly UL7 was shown to induce both myelopoiesis and monocyte differentiation. UL7 is also required for HCMV reactivation as neither CD34+ HPCs nor huNSG mice infected with UL7-deficient HCMV were able to reactivate from latency [188]. Since differentiation of early myeloid cells, such as CD34+ HPCs, infected with HCMV can trigger reactivation [190], UL7′s function as a differentiation factor explains its importance for reactivation [188].

Just as EGFR signaling is important to both betaherpesvirus tropism and latency, it also plays a role in viral reactivation. HCMV protein UL135 was previously proven to be required for reactivation, partially by decreasing total and cell surface EGFR levels and partially by overcoming the aforementioned latency-associated UL138 protein, which suppresses viral replication [191]. UL135 was reexamined in order to better understand the mechanism by which it controls reactivation. Using immunoprecipitation followed by tandem mass spectrometry (IP/MS) and yeast two-hybrid (Y2H) screen, UL135 was shown to interact with Abelson-interacting protein-1 (Abi-1) and Src homology 3 (SH3) domain-containing kinase binding protein 1 (SH3KBP1, also known as CIN85), two host adaptor proteins and signaling regulators. These interactions occur via polyproline sites, and disruption of these sites revealed that the interactions between UL135 and the two adaptor proteins modulate EGFR levels and intracellular EGFR trafficking. Finally, the interactions between UL135 and both Abi-1 and CIN85 are required for reactivation since infection with mutant viruses disrupting these interactions resulted in decreased reactivation [192].

As described earlier, HCMV produces several small regulatory RNAs known as miRNAs. HCMV miR-US22 affects EGR1, a transcription factor activated by EGFR signaling which regulates CD34+ HPC stemness. Specifically, miR-US22 has been demonstrated to downregulate EGR1 expression in HEK293 cells. This finding was confirmed by infection of NHDF cells with a miR-US22-deficient HCMV virus, which caused a marked increase in cellular EGR1 expression. Consistent with its effect on EGR1, miR-US22 also decreased CD34+ HPC proliferation. Finally, miR-US22 was found to be required for reactivation from latency as miR-US22-deficient viruses infecting CD34+ HPCs were unable to reactivate and produce infectious progeny [193].

In addition to its previously discussed role in maintaining latency through its interaction with NAB1, HCMV miR-US5-2 is critical in reactivation. Another target of miR-US5-2 is the EGFR adaptor protein GAB1, which activates PI3K and MAPK/ERK kinase (MEK)/ERK signaling. miR-US5-2 downregulates GAB1 expression and subsequently blocks EGFR-mediated PI3K and MEK/ERK signaling. These signaling pathways are both important for cell survival and proliferation, and miR-US5-2 expression accordingly was reported to decrease cell proliferation since miR-US5-2 and a GAB1 siRNA similarly blocked cell proliferation. The interaction between miR-US5-2 and GAB1 was further shown to influence downstream effects of EGFR signaling since EGR1 expression in cells transfected with miR-US5-2 was reduced following EGF stimulation relative to control cells. miR-US5-2 also regulates the expression of UL138, an HCMV protein important for latency, by targeting GAB1 and modulating EGR1 levels. This data suggests that miR-US5-2 plays a role in reactivation through the downregulation of GAB1, thereby reducing proliferation and UL138 expression [194].

miRNA regulation of TGF-β signaling, as discussed in the section on maintaining latency, is also involved in reactivation. While miR-US5-2 induces TGF-β signaling during HCMV latency, miR-UL22A blocks the TFG-β signaling pathway by decreasing expression of the TGF-β-responsive transcript SERPINE. miR-UL22A also targets SMAD3, which is required for latency in CD34+ HPCs. Infection with a miR-UL22A-deficient HCMV virus resulted in increased SMAD3 expression in NHDF cells and restored SERPINE transcript levels in HPCs, indicating that miR-UL22A impedes TGF-β signaling. Infection of CD34+ HPCs with miR-UL22A-deficient virus also resulted in a lower frequency of reactivation relative to wild type virus, while infection with miR-UL22A-deficient/SMAD3 shRNA virus restored reactivation potential. This again suggests that miR-UL22A is essential for HCMV reactivation. HCMV thus encodes two miRNAs, miR-US5-2 and miR-UL22A, which have antagonistic effects in order to have better control over TGF-β signaling [179].

HCMV reactivation was long assumed to be primarily controlled by de-repression of the major immediate early promoter (MIEP) (reviewed in [195]), but recent research has suggested otherwise. Collins-McMillen et al., reported that the re-expression of UL123 (encoding IE1 protein) and UL122 (IE2), which are both vital to reactivation, was associated with low levels of MIEP-derived transcripts following a reactivation stimulus in monocytic THP-1 cells. This suggests that IE1 and IE2 reactivation must be driven by alternative promoters and not MIEP. Indeed, transcripts of two intronic promoters (iP1 and iP2) 3′ of the MIEP were found to predominate in THP-1 cells following reactivation stimulus. Infection of THP-1 cells with a mutant virus lacking iP1 and/or iP2 failed to accumulate IE proteins and undergo reactivation, and CD34+ HPCs infected with mutant virus similarly displayed defects in reactivation, indicating that iP1 and iP2 are required for efficient reactivation of HCMV. In summary, this suggests that HCMV reactivation is controlled by multiple promoters and not solely MIEP [196].

To further elaborate on the findings that MIEP may not be as significant for HCMV reactivation as previously thought, the same lab investigated the mechanisms by which alternative promoters iP1 and iP2 operate. This report stated that forkhead family (FOXO) transcription factors are vital for the activation of iP1 and iP2 during reactivation. Mutation of FOXO binding sites in the alternative promoter iP2 decreased IE gene expression in THP-1 cells after reactivation stimulus. There was also a significant reduction in infectious virus production following reactivation in CD34+ HPCs infected with HCMV containing a mutated FOXO binding site. While not required, this suggests that FOXO binding sites within alternative intronic promoters are important for HCMV reactivation [197].

However, a different group saw contrasting results in dendritic cells that indicated that MIEP is indeed important for HCMV reactivation. Mason et al., [198] repeated the experiments done by Collins-McMillen et al., [196] and observed high levels of both iP2- and MIEP-derived IE gene transcription in THP-1 cells rather than high levels of iP1- and iP2-derived expression and low levels of MIEP-derived expression. Mason et al., further found that MIEP-derived transcription predominated in DC reactivation and also noted diverging levels of MIEP- and iP2-derived transcription in CD14+ monocytes stimulated down different differentiation pathways. This supported the thought that the promoters driving IE gene expression is affected by cell type and ligand-specific activity. iP2-derived transcription of IE genes was found to be cycloheximide dependent in DCs while MIEP transcription was not. This study concluded that control of HCMV reactivation may be cell type-specific and that MIEP still plays a key role in reactivation [198].

As discussed earlier in regard to the establishment of latency, the expression and activity of the c-fos subunit of cellular transcription factor AP-1 is decreased by HCMV GPCR US28 during latency [186]. AP-1 is also involved in reactivation of HCMV infection. In CD34+ Kasumi-3 cells, treatment with the c-fos inhibitor T-5224 decreased viral production after reactivation stimulus, indicating that AP-1 activation is essential for efficient reactivation. Use of a mutant virus, in which the proximal AP-1 binding site in the major immediate early (MIE) enhancer was disrupted, demonstrated that AP-1 recruitment to this binding site is not required for lytic replication but is required for efficient reactivation. Finally, AP-1 recruitment to the promoter proximal site is necessary for MIE-driven expression originating from the distal promoter (dP), iP2, and MIEP, but not iP1 or other IE genes. In sum, the binding of AP-1 to the MIE enhancer is vital for efficient HCMV reactivation [199].

Another GPCR encoded by HCMV is the protein UL33, the function of which was unknown until recently. Both UL33 mRNA and protein are expressed during latency in HCMV-infected Kasumi-3 cells. Mutation of the UL33 G protein-coupling domain revealed that UL33 is not required for establishment or maintenance of latency but is important for efficient reactivation. Following reactivation stimulus in THP-1 cells, UL33 signaling induces MIEP-driven gene expression. UL33-induced signaling also activates cellular cyclic AMP response element binding protein (CREB1) and enables CREB1 recruitment to the MIE enhancer/promoter. Use of pharmacological CREB phosphorylation inhibitors confirmed that UL33′s regulation of CREB phosphorylation is the mechanism by which it affects reactivation [200].

Finally, a study analyzed how latent HCMV infection affects the secretome of CD14+ monocytes. IL-10, CCL8, and CXCL10 were all found to be significantly upregulated under these conditions. The secretome of CD14+ monocytes during HCMV latency was also discovered to promote CXCL10-mediated migration of activated, but not resting, CXCR3+ immune cells, including CD4+ T cells. The secretome of CD14+ monocytes additionally decreases antiviral activity of activated CD4+ T cells. However, co-culturing latently-infected CD14+ monocytes with activated CXCR3+ CD4+ T cells resulted in HCMV reactivation. These findings suggest that the secretome of latently-infected cells is modulated by HCMV, affecting the local microenvironment [201].

Little is currently known about reactivation of HHV-6 and HHV-7. Though HHV-6A and HHV-6B have been associated with several clinical conditions, the mechanisms by which these viruses reactivate remain elusive [17,202]. The HHV-6 putative integrase U94, mentioned above, is reported to be involved in latency, integration, and reactivation, though its mechanistic role is not well understood [202]. Some limited data regarding HHV-6 reactivation has been reviewed by Pantry et al., [203]. In sum, more research is needed in order to better understand the reactivation of HHV-6 and HHV-7.

## 6. Vaccination Efforts

Betaherpesviruses express numerous glycoproteins that serve as essential components of their fusion machinery [204,205,206]. Recently, these glycoproteins have been assessed as potential targets in vaccination efforts.

Of the betaherpesviruses, efforts to create a vaccine against human cytomegalovirus have been the most robust. As was mentioned previously, HCMV is associated with congenital infection and a relatively high risk of associated birth defects, including but not limited to cognitive impairment, cerebral palsy, bilateral hearing loss, and death [21,207]. HCMV infection also causes significant complications in transplant patients, such as transplant-associated vasculopathy, hepatitis, retinitis, infection, and graft rejection [208,209].

The HCMV glycoprotein B (gB) has been examined as a potential target for vaccination efforts. Nelson et al., recently examined a lipid nanoparticle vaccine (LNPV) containing a nucleoside modified mRNA transcript encoding full length gB protein in comparison to vaccines containing gB ectodomain (gB protein with antigenic domain 3 removed) with a squalene adjuvant and full length gB with an MF59-like squalene-based adjuvant. Three doses of each vaccine were delivered to New Zealand White rabbits four weeks apart. While the three vaccines were equivalent in their maximal immunogenicity and elicited IgG affinity for gB, the LNPV demonstrated enhanced response longevity relative to the other two. In addition, the LNPV was able to generate antibodies with a broader spectrum of gB peptide binding abilities [210].

A second effort at targeting gB for vaccine development found that HCMV-neutralizing antibodies primarily targeted antigenic domain 5 [211]. Using this information, a nanoparticle vaccine was created that increased titers of neutralizing antibodies by 100-fold in comparison to gB extracellular domain vaccine [211], presumably by increasing the number of copies of antigen presented and by presenting the antigens in a well-ordered manner that may more closely resemble the pathogen surface [211,212].

While gB is frequently targeted for HCMV vaccine generation, other promising targets have emerged. The gH/gL/UL128/UL130/UL131 pentamer, a glycoprotein complex required for entry into epithelial and endothelial cells but not fibroblasts [205], is one such target. A recent series of experiments by Chiuppesi et al., used a modified vaccinia virus ankara (MVA) vector to deliver an antigen including the pentamer complex, the strong T cell stimulator [213] phosphoprotein 65, and gB. The antigen stimulated a potent immune response involving both humoral and cell-mediated immunity [214]. A separate study using a guinea pig/guinea pig cytomegalovirus model demonstrated that a disabled infectious single-cycle viral vaccine expressing the pentamer complex was 97% effective in preventing congenital guinea pig cytomegalovirus infection [215]. Notably, the guinea pig is an accepted animal model for human congenital cytomegalovirus infection [216,217]. A more comprehensive review of cytomegalovirus vaccination principles and recent advances in HCMV vaccine technology can be found elsewhere [218,219,220].

While it has not received the same degree of attention as HCMV, there have been some recent efforts to develop a vaccine for HHV-6B. HHV-6 expresses several envelope glycoproteins, including glycoproteins H, L, Q1, and Q2, or gH, gL, gQ1, and gQ2, respectively [221,222]. These glycoproteins complex to form a gH/gL/gQ1/gQ2 tetramer that facilitates viral entry by binding CD46 during HHV-6A infection and CD134 during HHV-6B infection [223,224]. A recent study by Wang et al., assessed the efficacy of the gH/gL/gQ1/gQ2 tetramer in conjunction with the common vaccine adjuvants aluminum hydroxide gel adjuvant (alum) and D35, a CpG oligodeoxynucleotide adjuvant. N-[1-(2,3-Dioleoyloxy)propyl]-N,N,N-trimethylammonium methylsulfate was mixed with the D35. A series of three injections given to hSIRPα-DKO mice stimulated the development of both cell-mediated and humoral immunity as measured by cytokine production (specifically IFN-γ and IL-13) and antibody production, respectively [225]. A PubMed search did not reveal any research into developing a vaccine against HHV-7.

## 7. Concluding Remarks

Betaherpesviruses represent a highly successful family of viruses capable of modulating the human immune system to facilitate their survival. While the viruses making up the betaherpesvirus subfamily target many of the same systems, they have evolved unique methods of avoiding the immune system. In this review, we examined recent advances in the understanding of how betaherpesviruses interact with and evade the immune system ranging from avoiding the initial immune response to control of latency and reactivation. In addition, recent advances in vaccine technology were briefly discussed.

While HCMV has well-established disease associations, emerging data has begun to link HHV-6 and HHV-7 to other illnesses as well. For example, recent research discussed at the 11th International Conference on Human Herpesviruses-6A, -6B, and -7 indicated these viruses may play a role in the pathogenesis of neurodegenerative disorders including Alzheimer’s disease and multiple sclerosis [226], while other reports have linked these viruses to diseases such as depression [227] and epilepsy [228,229]. As more evidence comes to light and the need for effective treatment for these viruses becomes clear, a thorough understanding of the interplay between the immune system and betaherpesviruses becomes increasingly imperative.

## Figures and Tables

**Table 1:** Summary of Betaherpesvirus Immunoevasive strategies.

Virus	Immunoevasive Strategy	Associated References
**HCMV**	UL35: Decrease TBK/IRF3 phosphorylation	[61]
	UL42: Inhibit cGAS’ ability to bind HCMV DNA Inhibit cGAS oligomerization Facilitate TRAPβ degradation (lysosome)	[56]
	UL94: Inhibit STING dimerization Prevent STING from recruiting TBK1	[57]
	IE86: Facilitate STING degradation via proteasome	[58,59]
	US7: Facilitate TLR3 and TLR4 proteasomal degradation	[69]
	US8: Inhibit TLR3 and TLR4 by destabilization	[69]
	IE1: Suppress CpG motifs to avoid ZAP detection	[81]
	UL26: Inhibit ISG15/ISGylation	[85]
	pUL145: Downregulate STAT	[90]
	UL147a: Target MICA for lysosomal degradation	[102]
	UL148a: Target MICA for lysosomal degradation	[101]
	gB: Interact with epidermal growth factor receptor to stimulate the AKT pathway	[157,158,159]
	gH: Interact with αvβ3 integrin to stimulate the AKT pathway	[157,158,159]
	pUL7: Stimulate phosphorylation, translocation to cytoplasm, and inactivation of FOXO3a	[162]
	miR-US5-1 and miR-UL112-3p: FOXO3a inhibition	[162]
	Downregulate CIITA	[117]
	Downregulate MHC class I expression	[122]
	Alter expression of apoptotic genes	[150]
**HHV-6A**	Downregulate IFI16	[62]
	Induce phosphorylation of STAT6	[62]
	Inhibit TLR9 mRNA and protein expression	[62]
	Infect NK cells and alter miRNA and transcription factor expression	[24]
	Modulate HLA class I and class II expression	[130]
	Alter T lymphocyte miRNA upon infection	[131]
	Favor TH2 cytokine profile upon dendritic cell infection	[145]
	Alter expression of apoptotic genes	[150]
	Increase autophagic flux	[165]
	Increase BiP	[165]
**HHV-6B**	Downregulate IFI16	[62]
	Inhibit TLR9 protein levels	[62]
	Inhibit TLR signaling	[70]
	Inhibit autophagy	[165,167]
	Upregulate CHOP via modulating expression of IRE1α, ATF4, and ATF6	[165]
	Inhibit monocyte survival and differentiation into dendritic cells	[167]
	Increase ROS to upregulate PD-L1, influence STAT phosphorylation	[93]
	Downregulate NKG2D ligands	[106]
	Infect NK cells and alter miRNA and transcription factor expression	[24]
**HHV-7**	U21: Targets MCH class I complex for lysosomal degradation, target UBPL1 for lysosomal degradiation, decrease MICA and MICB concentration, post-transcriptional modification of MICA and MICB	[109,110,111,133]
	Slight decrease in cGAS expression	[62]

## Data Availability

Not available.
